# Association between common respiratory pathogens and disease severity, and pathogen-specific seasonality in the Caribbean pre-COVID-19 and post-COVID-19: a retrospective study

**DOI:** 10.1136/bmjopen-2025-104991

**Published:** 2026-01-07

**Authors:** Sam Engels, Martijn Tilanus, Juldany Juliet, Marquita Euson, Nika Stastny, Charlene Maria, Sharda Baboe-Kalpoe, Fazal Baboe, Chérina K A Fleming, Sherryl Carty-Fleming, Shanna Holaman, Kaylee Baan, Felix Holiday, Sebastiaan C F Van den Borne, Rutger F Plantinga, Dick Wong Chung, Josephine van de Maat, Marien I de Jonge, Radjin Steingrover, Lilly M Verhagen

**Affiliations:** 1 Laboratory of Medical Immunology, Radboudumc, Nijmegen, Netherlands; 2 St. Maarten Medical Center, Cay Hill, Sint Maarten; 3 St. Eustatius Health Care Foundation, Oranjestad, Sint Eustatius and Saba, Netherlands; 4 St. Maarten Laboratory Services, Cay Hill, Sint Maarten; 5 Ear, Nose, Throat Department, Canisius Wilhelmina Ziekenhuis, Nijmegen, Netherlands; 6 De Vijf Meren Kliniek, Haarlem, Netherlands; 7 Department of Pediatric Infectious Diseases and Immunology, Amalia Children's Hospital, Radboudumc, Nijmegen, Netherlands; 8 Fundashon Mariadal, Kralendijk, Bonaire, Netherlands

**Keywords:** Respiratory infections, PAEDIATRICS, Infection control

## Abstract

**Abstract:**

**Introduction:**

Respiratory tract infections (RTIs) cause significant child morbidity and mortality. Periodical influenza vaccination and respiratory syncytial virus (RSV) prophylaxis can reduce this burden in risk groups. However, in the Caribbean, the optimal timing of these interventions is unclear due to a lack of epidemiological data. We aimed to investigate pathogens associated with RTI disease burden and pathogen specific seasonality in the Caribbean in the context of COVID-19 to achieve optimal timing of preventive measures.

**Methods:**

We conducted a retrospective study using patient records and pathogen detection data from St. Maarten Medical Center from 1 September 2018 to 1 September 2023. We performed regression to associate pathogens with outcomes and seasonality.

**Results:**

RTI diagnoses accounted for 50.8% (N=7380) of outpatient cases and 28.0% (N=508) of inpatient cases. RSV and rhino/enterovirus were associated with more frequent oxygen requirement (OR 5.1 (95% CI 2.3 to 11) and OR 2.3 (95% CI 1.2 to 4.3), respectively) and tachypnoea/dyspnoea (OR 4.9 (95% CI 2.0 to 13) and OR 2.8 (95% CI 1.6 to 5.2), respectively) than other pathogens post-COVID-19. RSV consistently peaked during June/July and September/October, preceding RSV prophylaxis administration in October.

**Conclusions:**

The overall burden on the healthcare system due to RTI visits and admissions was high. Higher disease severity was associated with RSV and rhino/enterovirus infections; therefore, universal RSV prophylaxis should be considered, and timing should be optimised based on seasonality.

**STRENGTHS AND LIMITATIONS OF THIS STUDY**

- A 5-year dataset (2018–2023) spanning both the pre-COVID-19 and post-COVID-19 periods, enabling multiyear seasonality and comparative analyses.
- The use of the BioFire Respiratory Panel allowed for detection of a wide variety of viral pathogens, including six known major contributors to disease burden in children.
- Multivariable models were fitted that adjusted for key clinical covariates to reduce confounding.
- Pathogen-specific data were only available for inpatients, limiting the generalisability of the results to outpatients.
- Modest sample size, increasing the risk of type II error and reducing power for subgroup analyses.

## Introduction

 Globally, respiratory tract infections (RTIs) are a major contributor to disease burden in children.^1^ Especially lower RTIs (LRTIs) are one of the leading causes of death, particularly in children under the age of 5, accounting for 672 000 deaths in 2019 worldwide and roughly a third of paediatric hospitalisations.^1^,^3^ Child malnutrition is an important risk factor, contributing to 53%–56% of LRTI mortality, alongside other risk factors such as household air pollution, low birth weight and inadequate hand hygiene practices.^1^ Beyond mortality, childhood LRTIs can result in long-term morbidity, including impaired lung function and a two times increased risk of mortality from respiratory disease in adulthood.^4^

In particular, observational studies have found an association between respiratory syncytial virus (RSV) LRTI in early life and ongoing respiratory morbidity, including recurrent wheezing and asthma within the first decade.^5^ However, it is possible that these findings primarily indicate RSV LRTI in early life as a marker for an underlying predisposition to such respiratory conditions.^6^ Overall, viral pathogens contribute substantially to the global disease burden and were found in over 30% of pneumonia-related deaths in children.^7^ In addition to RSV, other viral causes of severe LRTIs, including rhinovirus, influenza and human metapneumovirus (HMPV), lead to hospitalisations.^7^,^9^ Of all viral pathogens, RSV and influenza cause the highest disease burden globally in children under 5 years.^10^

To mitigate this burden, the WHO advocates preventive measures against RSV and influenza, including RSV prophylaxis with monoclonal antibodies for high-risk groups and seasonal influenza vaccination, with the timing of administration depending on the seasonality of these pathogens.^11^,^13^ Recently, long-acting monoclonal antibodies for RSV have become available, allowing universal protection. RSV and influenza have a consistent peak in winter in most temperate climates, while seasonality has been more variable in (sub)tropical climates, leading to country-specific suggestions for influenza vaccination timing in these regions, either in April or October.^12 14^ Nonetheless, in some countries, vaccination and prophylaxis schedules may not align with local climate patterns in practice. In particular, on several islands in the Caribbean part of the Kingdom of The Netherlands, the timing for influenza vaccination and RSV prophylaxis is set to September–October, following the guidelines of the Netherlands. This may not be appropriate given the climatic differences between countries and is also inconsistent with WHO recommendations for vaccination in April in the Caribbean.^14^ However, the true seasonality of these pathogens in the Caribbean remains unclear because, as in most studies on the seasonality of RSV and influenza in the Caribbean, the data are combined with those of other countries in Latin America, while the climate varies greatly within this region.^15^

Understanding viral transmission dynamics on islands is important due to the unique environmental factors and population dynamics that can influence disease spread. Islands often have distinct patterns of human movement compared with mainland areas. These factors can affect the seasonality and intensity of virus outbreaks, as well as the effectiveness of preventive measures. Additionally, tourism leads to fluctuations in population size and composition throughout the year, and seasonality in the Caribbean part of the Kingdom of the Netherlands may be heavily influenced by the considerable number of tourists from temperate regions, with over 2 million tourist visits per year in the entire region for a local population of 300–400 thousand inhabitants.^16 17^

The effectiveness of preventive measures relies on an accurate understanding of the burden of disease. This has been insufficiently studied in the Caribbean, partly due to limited access to pathogen-specific diagnostic tools.^18^ Considering that the disease burden of LRTIs in children may have increased globally after the COVID-19 pandemic,^19^,^22^ it is particularly important to evaluate this burden both before and after the COVID-19 pandemic. To gain more insight into the burden of RTIs in the Caribbean region and the optimal timing for preventive measures, we investigated the total number of affected patients and disease severity among children with RTIs in a hospital in the Caribbean part of the Dutch Kingdom. Furthermore, we investigated the pathogens associated with disease severity and their seasonality in the context of the COVID-19 pandemic. Specifically, our aims were: (1) to quantify paediatric RTI disease burden at St. Maarten Medical Center, describing care utilisation for inpatients and outpatients; (2) to assess, among inpatients with BioFire testing, the associations between individual respiratory pathogens and indicators of disease severity, using multivariable and age-stratified analyses; (3) to characterise the seasonality of pathogens associated with severity among inpatients, and to consider implications for the timing of preventive strategies when available and (4) to describe associations between respiratory infection syndromes and participant or clinical characteristics, and to compare these patterns across the pre-COVID-19 and post-COVID-19 periods.

## Methods

### Study setting

For this retrospective observational study, we used electronic patient records from the SMMC between 1 September 2018 and 1 September 2023. St. Maarten, situated as an independent nation within the Kingdom of the Netherlands in the Caribbean, was chosen as the study site due to its central location and status as referral centre for children with (severe) LRTIs from two neighbouring islands, Saba and St. Eustatius.

### Patient and public involvement

The patient/public were not involved in the design of the study, as this was deemed not appropriate for the nature of this research as an observational study.

### Study population and definitions

The study included children aged <18 years who sought care at the SMMC for respiratory symptoms. Outpatients were defined as patients who had visited the SMMC with symptoms but had not been admitted, whereas inpatients were defined as all patients admitted to the hospital due to an RTI. RTI diagnosis identification for outpatients and inpatients was based on a set of International Classification of Diseases (ICD-10) codes related to RTIs (online supplemental file 1), and children were included if they presented with at least one RTI symptom (cough, wheezing, abnormal auscultation, dyspnoea, tachypnoea and upper respiratory symptoms). Missing documentation regarding discharge was a reason for exclusion. Inpatients were systematically divided into four diagnostic categories by two research members; (1) upper RTIs (URTI), defined as the presence of at least one respiratory symptom without tachypnoea or respiratory distress; (2) LRTI, defined as the presence of at least one respiratory symptom with tachypnoea or respiratory distress, including bronchiolitis or pneumonia; (3) viral wheeze, defined as wheezing as predominant clinical presentation below the age of six and (4) asthma exacerbation, defined as wheezing as predominant clinical presentation from age six and older. Seasonality was defined as the collective period in which all peak months during the study period occurred, with peak month defined as months in which more cases occurred than the 75th percentile of cases per month. Pathogen presence was defined as positive detection of a pathogen by the BioFire Respiratory Panel (BioFire), which identifies respiratory pathogens directly from patient samples using multiplex PCR. The introduction of the BioFire Respiratory Panel as a diagnostic tool in St. Maarten in September 2018 limited the availability of pathogen-specific diagnoses before this date, leading to the exclusion of patients admitted before 1 September 2018.

### Data collection

Anonymised data were retrieved from electronic patient records and entered into a Castor database. Demographic information (eg, sex, age, date of admission), BioFire results (ie, adenovirus, coronavirus (229E, HKU1, NL63, SARS-CoV-2), rhino/enterovirus, HMPV, influenza (A, AH1, AH12009, B), parainfluenza (type 1–4), RSV, *Bordetella pertussis*, *Bordetella parapertussis*, *Chlamydia pneumoniae*, *Mycoplasma pneumoniae*), diagnoses (URTI, LRTI, viral wheeze, asthma exacerbation), symptoms (eg, dyspnoea, tachypnoea) and clinical characteristics (eg, oxygen use at admission, length of admission) were collected (online supplement 2).

### Statistical analysis

To assess overall RTI burden expressed in numbers and percentage of children admitted to the hospital for RTI each year, all outpatient and inpatient data were used. In the inpatient cohort, we conducted descriptive statistics to describe patient characteristics and the distribution of pathogens, using frequencies and percentages for categorical variables, and median and IQR for non-normally distributed continuous variables. Additionally, we performed regression analyses to assess the association between presence of pathogens and diagnoses as well as clinical severity. For clinical severity, we considered tachypnoea and/or dyspnoea and oxygen use as indicators for severity of disease. In addition, we performed regression analysis to assess the association between presence of pathogens and occurrence during either the dry or rainy season as a binary variable, with pre-COVID-19 or post-COVID-19 pandemic included as a confounding variable. Only the subset of patients with a positive BioFire and thus available pathogen data were included in regression analyses, and for each regression analysis, only pathogens that had at least one patient positive for the outcome were included. The date of admission was used to infer whether an admission had occurred during the dry or rainy season, where dry season ranged from 1 December until 30 April and rainy season from 1 May to 30 November.^23^ Wilcoxon rank-sum test and χ² test were used to assess the association between patient characteristics and pre-COVID-19 and post-COVID-19, for continuous and binary variables, respectively. Regression was used to assess the association between severity outcomes and period (pre-COVID-19 and post-COVID-19), where linear regression was used for length of hospital stay and logistic regression for all other outcomes. Patients were divided into a pre-COVID-19 and post-COVID-19 group based on their date of admission, with a cut-off date of March 29 2020 as this marked the first official lockdown measures in St. Maarten. Additionally, we performed subset analyses on all outcomes for young children (aged ≤2 years) and older children (aged >2 years).

Assumptions for logistic and linear regression were examined using a VIF score for multicollinearity and inspection of the distribution of residuals before running the statistical models. For logistic regressions, adjusted ORs including 95% CI were calculated. Pathogen subtypes were pooled if they shared a common pathogen group, such as coronavirus (229E, HKU1, NL63, SARS-CoV-2), influenza (A, AH1, AH12009, B) and parainfluenza (type 1–4). Pathogens were excluded from further analysis if the pooled group showed an overall prevalence lower than 5% of the total positive cases. Additionally, pathogens were excluded from individual analyses if the pathogen had no cases for a particular clinical outcome. As missingness of outcome measures was negligible in the dataset, no additional models were employed to compensate for missing values. We used R Statistical Software (V.4.1.3; R Core Team 2022) for all analyses. A p value <0.05 was considered statistically significant.

## Results

**Figure 1:**
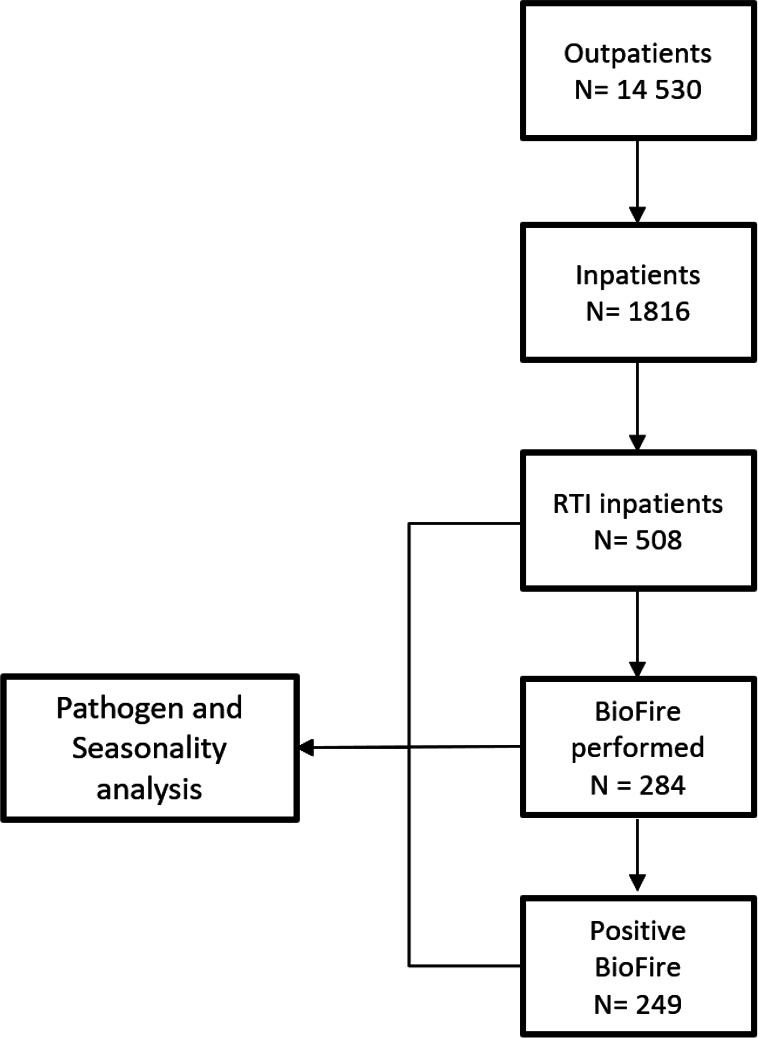
Flow diagram of patient cohorts used per analysis. RTI, respiratory tract infection.

During the study period, there were 14 530 paediatric outpatient visits (figure 1). Among these, 7380 children (50.8%) were diagnosed with an RTI. Among the inpatient cohort of 1816 children, 508 (28%) were admitted due to an RTI. Within this group, nine patients (1.8%) were admitted to the paediatric intensive care unit (PICU), and one child (0.2%) died. The proportion of RTI patients relative to all presenting patients ranged between 51%–67% for outpatients and 23%–38% for inpatients in 2018, 2019, 2022 and 2023. However, in 2021 and 2021, there was a notable decrease, with only 38%–40% of outpatient and 15%–20% of inpatient diagnoses attributed to an RTI (online supplemental 3). Although the proportion of RTI patients compared with total patients was fairly similar for outpatients and inpatients in the first 2 years of the study, there was a noticeable increase in outpatients in 2022 and 2023, (66.9% and 65.0%, respectively), while the proportion of RTI inpatients returned to prepandemic levels (online supplement 3).

Further analyses only included the inpatient population of 508 children with RTIs, with a mean age of 2.7 years (table 1). Most patients (63.6%) were male, and 55.9% underwent BioFire testing at admission. LRTI was the most common diagnosis in young children (45.3%), followed by URTI and viral wheeze (29.3% and 25.7% respectively), with no cases of asthma exacerbation as by definition this could only occur in older children. On the other hand, older children most commonly experienced viral wheeze (32.0%), followed by URTI and LRTI (25.9% and 25.4%) and finally asthma exacerbation (16.2%). In young children, the most common pathogen was rhino/enterovirus, constituting 56.0% of all detected pathogens, followed by RSV (33.7%), adenovirus (13.3%) and parainfluenza (13.3%). In older children, however, the two most common pathogens were rhino/enterovirus (74.7%) and influenza (10.8%) (online supplemental file 4a). All other pathogens made up less than 10% of detections each. Coinfections were observed in 26.7% of cases, notably higher in children with adenovirus (86%) and parainfluenza (65%), and lower in children with rhino/enterovirus (32%) and influenza (5%). These differences were statistically significant when pathogens were compared individually in pairs, reflecting that the proportion of coinfection was higher in cases with adenovirus or parainfluenza than in those with rhino/enterovirus or influenza (p<0.01). Mean age was highest in children with *Mycoplasma pneumoniae* (7.0 years) and influenza (3.7 years), and lowest in children with parainfluenza (1.2 years, p=0.03) and RSV (0.93 years, p<0.01). Overall, 15.1% of individual patients experienced multiple admissions due to RTIs during the study period, of which 4.2% experienced three or more. In the subset of multiple admissions, the most common pathogen was rhino/enterovirus (73.0%) followed by RSV (18.9%), adenovirus and influenza (8.1%), and HMPV and parainfluenza (5.4%).

**Table 1:** Patient characteristics

Variable	Total	Pre-COVID-19	During/post-COVID-19	OR/(95% CI)	P value
	N=508	N=245	N=263		
	508	245	263		
Gender male—N (%)	323 (63.6)	162 (66.1)	161 (61.2)	0.81 (0.56 to 1.2)	0.25
Age (years)—Median (IQR)	2.0 (0.75–4.0)	2.0 (0.0–4.0)	2.0 (1.0–4.0)	(–8.6 to 1.7)	0.82
BioFire performed—N (%)	284 (55.9)	72 (29.4)	212 (80.6)	9.9 (6.6 to 15.1)	2.2E–16*
Positive Biofire—N (%)	249 (49.0)	63 (25.7)	186 (70.7)		
Diagnosis					0.33
LRTI—N (%)	191 (37.6)	95 (38.8)	96 (36.5)	0.91 (0.63 to 1.3)	
URTI—N (%)	142 (28.0)	76 (31.0)	66 (25.1)	0.75 (0.50 to 1.1)	
Viral Wheeze—N (%)	143 (28.2)	61 (24.9)	82 (31.2)	1.4 (0.93 to 2.0)	
Asthma exacerbation—N (%)	32 (6.3)	15 (6.1)	17 (6.5)	1.1 (0.51 to 2.2)	
Number of pathogens—Median (IQR)	1.0 (1.0–1.0)	1.0 (1.0–1.0)	1.0 (1.0–1.0)	(-8.0 to 1.9)	0.29
1 pathogen—N (%)	185 (74.3)	51 (81.0)	134 (72.0)		
2 pathogens—N (%)	54 (21.7)	10 (15.9)	44 (23.7)		
3 or more pathogens—N (%)	10 (4.0)	2 (3.2)	8 (4.3)		
Pathogen					
Rhino/enterovirus—N (%)	155 (62.3)	37 (58.7)	118 (63.4)	1.2 (0.67 to 2.2)	0.51
RSV—N (%)	64 (25.7)	19 (30.2)	45 (24.2)	0.74 (0.39 to 1.4)	0.35
Adenovirus—N (%)	30 (12.1)	4 (6.4)	26 (14.0)	2.3 (0.85 to 8.3)	0.11
Parainfluenza—N (%)	26 (10.4)	9 (14.3)	17 (9.1)	0.60 (0.26 to 1.5)	0.25
Influenza—N (%)	19 (7.6)	4 (6.4)	15 (8.1)	1.3 (0.43 to 4.7)	0.66
HMPV—N (%)	16 (6.4)	3 (4.8)	13 (7.0)	1.4 (0.44 to 6.8)	0.53
SARS-CoV-2—N (%)	12 (4.8)	0 (0.0)	12 (6.5)	NA	0.039
Mycoplasma pneumoniae—N (%)	2 (0.8)	1 (1.6)	1 (0.5)	0.34 (0.01 to 13)	0.42
Disease severity					
Oxygen use—N (%)	101 (19.9)	40 (16.3)	61 (23.2)	1.5 (1.0 to 2.4)	0.05
Dyspnoea/Tachypnoea—N (%)	323 (63.6)	153 (62.5)	170 (64.4)	1.1 (0.76 to 1.6)	0.61
Length of admission—Median (IQR)	2.0 (1.0–3.0)	3.0 (2.0–4.0)	2.0 (1.0–3.0)	(1.0 to 1.0)	8.4E–11*
Season of admission					
Rainy season—N (%)	230 (45.3)	111 (45.3)	119 (45.3)	1.0 (0.70 to 1.4)	0.99
Dry season—N (%)	278 (54.7)	134 (54.7)	144 (54.8)		
Antibiotic prescription—N (%)	236 (46.5)	141 (57.6)	95 (36.1)	0.42 (0.29 to 0.60)	1.3E–6*

P values are based on t-test for normally distributed variables and Wilcoxon rank sum test for non-normally distributed variables. ORs and 95% CIs are provided for categorical variables, and 95% CI is provided for continuous variables.

* Statistically significant.

HMPV, human metapneumovirus; LRTI, lower respiratory tract infection; NA, not available; RSV, respiratory syncytial virus; URTI, upper RTI.

Comparing pre-COVID-19 and post-COVID-19 subsets of admissions, patient characteristics and diagnoses did not differ significantly (table 1). The percentage of BioFire tests performed did change, increasing from 29.4% pre-COVID-19 to 80.6% post-COVID-19. Notable differences in pathogen distribution included the percentage of adenovirus, which increased from 6.4% to 14.0% (p=0.11), SARS-CoV-2, which increased from 0% to 6.5% (p=0.039), and parainfluenza, which decreased from 14.3% to 9.1% (p=0.25) pre-COVID-19 and post-COVID-19, respectively. Notably, these changes mostly reflect trends in young children, whereas in older children, only adenovirus showed an increase from 0.0% to 12.7% whereas other pathogens remained stable. Length of admission showed a significant decrease, from 3.2 days pre-COVID-19 to 2.4 days post-COVID-19. Lastly, the percentage of patients receiving antibiotics was significantly lower post-COVID-19 (57.6 to 36.1%, p<0.01).

Pathogens excluded from further analyses were SARS-CoV-2 and *Mycoplasma pneumoniae*, as their prevalence throughout the whole study period was considered too low in our study sample (respectively 4.8 and 0.8%).

### Pathogens, seasonality and clinical outcomes

URTI was significantly more commonly diagnosed in children with influenza than in those with other pathogens and in males compared with females, and significantly less common in children with RSV, parainfluenza and rhino/enterovirus (table 2a). On the other hand, LRTI was more common in children with RSV and HMPV, and also more common in the rainy season, older children and males (table 2b). Finally, viral wheeze was significantly more common in children with rhino/enterovirus, whereas it was less common in children with RSV and older children, and in the rainy season (table 2c). Asthma exacerbation could not be tested due to low numbers.

**Table 2:** Linear and logistic regression models of the association between the different pathogens and (a) URTI diagnosis, (b) LRTI diagnosis, (c) viral wheeze diagnosis, (d) oxygen requirement, (e) dyspnoea/tachypnoea

(a) URTI	OR	95% CI	P value	(d) Oxygen requirement	OR	95% CI	P value
Adenovirus	1.8	0.70 to 4.6	0.20	Adenovirus	1.2	0.49 to 2.9	0.64
Rhino-Enterovirus	0.45	0.27 to 0.73	1.8E–3*	HMPV	1.3	0.33 to 4.3	0.66
Influenza	6.9	2.4 to 25	9.9E–4*	Rhino-Enterovirus	2.3	1.4 to 3.8	5.1E–4*
Parainfluenza	0.12	0.01 to 0.63	0.045*	Parainfluenza	1.1	0.36 to 2.8	0.91
RSV	0.10	0.02 to 0.29	2.1E–4*	RSV	4.2	2.2 to 7.7	5.4E–6*
Rainy season	0.87	0.57 to 1.3	0.54	Rainy season	1.2	0.77 to 2.0	0.39
Age (years)	0.97	0.91 to 1.0	0.50	Age (years)	1.1	1.0 to 1.2	2.4E–3*
Male gender	1.6	1.0 to 2.4	0.04*	Male gender	1.1	0.65 to 1.7	0.82

(b) LRTI	OR	95% CI	P value	(e) Tachypnoea/Dyspnoea	OR	95% CI	P value
Adenovirus	0.86	0.35 to 2.0	0.74	Adenovirus	0.65	0.28 to 1.5	0.31
HMPV	3.4	1.2 to 10.6	0.03*	HMPV	2.6	0.80 to 12	0.15
Rhino-Enterovirus	1.0	0.67 to 1.6	0.86	Rhino-Enterovirus	2.0	1.3 to 3.1	2.2E–3*
Influenza	0.53	0.12 to 1.7	0.33	Influenza	0.27	0.09 to 0.75	0.017*
Parainfluenza	1.6	0.67 to 4.0	0.29	Parainfluenza	1.1	0.44 to 3.0	0.86
RSV	6.8	3.6 to 14	1.9E–8*	RSV	3.6	1.8 to 8.0	6.0E–4*
Rainy season	1.6	1.1 to 2.4	0.03*	Rainy season	0.93	0.63 to 1.0	0.70
Age (years)	0.88	0.81 to 0.95	2.3E–3*	Age (years)	0.96	0.90 to 1.0	0.24
Male gender	0.55	0.35 to 0.84	6.5E–3*	Male gender	0.62	0.42 to 0.92	0.019*

(c) Viral wheeze	OR	95% CI	P value
Adenovirus	0.78	0.31 to 1.8	0.57
HMPV	1.3	0.43 to 3.7	0.62
Rhino-Enterovirus	1.6	1.0 to 2.4	0.04*
Influenza	0.13	0.01 to 0.67	0.05
Parainfluenza	1.4	0.55 to 3.2	0.49
RSV	0.47	0.22 to 0.91	0.03*
Rainy season	0.60	0.40 to 0.91	0.02*
Age (years)	0.93	0.86 to 1.0	0.046*
Male gender	1.2	0.81 to 1.9	0.34

* Statistically significant.

HMPV, human metapneumovirus; LRTI, lower respiratory tract infection; RSV, respiratory syncytial virus; URTI, upper RTI.

Two-thirds of children presented with dyspnoea and/or tachypnoea, and 20% of children needed oxygen therapy, whereas 177 (34.8%) had neither oxygen requirement nor dyspnoea/tachypnoea. Rhino/enterovirus, RSV and age showed significant positive associations with the need for oxygen treatment during admission, with ORs of 2.3 (95% CI 1.4 to 3.8), 4.2 (95% CI 2.2 to 7.7), and 1.1 (95% CI 1.0 to 1.2, p=2.4E-3), respectively (table 2d), which was similar in young and older children, although the association with rhino/enterovirus was stronger in older children (online supplemental file 4b). Regarding tachypnoea and/or dyspnoea, rhino/enterovirus and RSV showed statistically significant positive associations, with ORs of 2.0 (95% CI 1.3 to 3.1) and 3.6 (95% CI 1.8 to 8.0), respectively. However, this association with RSV was only present in young children (online supplemental file 4b). Conversely, tachypnoea was significantly less commonly associated with influenza infection and in males, with an OR of 0.27 (95% CI 0.09 to 0.75) and 0.62 (95% CI 0.42 to 0.92) (table 2e), respectively. Associations did not change when incorporating length of admission or coinfection in the model as confounders.

### Pre-COVID-19 and post-COVID-19 comparison of clinical outcomes

**Figure 2:**
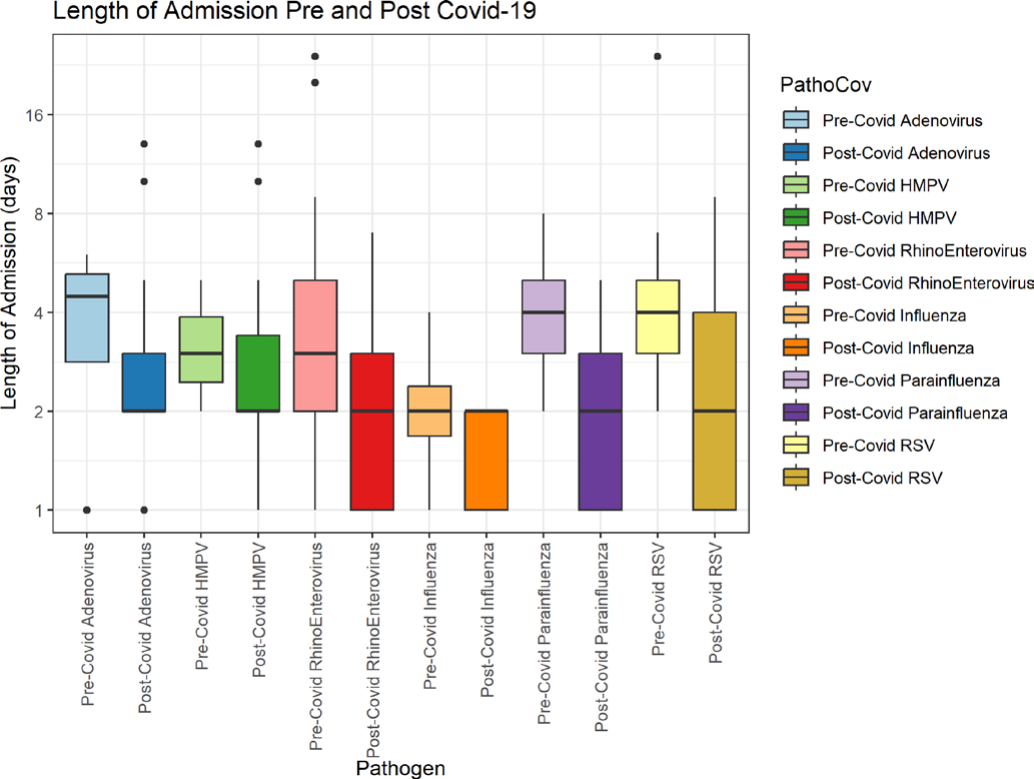
Boxplot of hospital admission length for all pathogens divided by pre-COVID-19 and post-COVID-19. HMPV, human metapneumovirus; RSV, respiratory syncytial virus.

Overall, oxygen requirement and tachypnoea/dyspnoea occurrence did not significantly differ pre-COVID-19 and post-COVID-19 (16.3% to 23.2%, p=0.05, and 62.5% to 64.4%, p=0.61, respectively). Remarkably, length of admission showed a significant decrease post-COVID-19 both in the total inpatient population and the subset of patients tested with BioFire, but not in patients who were not tested with BioFire. This decrease was supported by visualisation separated by pathogen (figure 2). Indeed, additional analysis revealed that admission length was statistically significantly associated with pre-COVID-19 or post-COVID-19 as a covariate, with an estimate of −1.1 days (95% CI −1.5 to −0.61, p=4.8E−6).

We performed sensitivity analyses to assess the generalisability between subsets. For the patient subset tested with the BioFire panel, no statistically significant differences in outcomes were observed pre-COVID-19 and post-COVID-19, except the aforementioned decrease in length of admission (from 4.2 to 2.5 days, p<0.01) (full table in online supplemental file 5a). Additionally, we compared the BioFire population to the total population pre-COVID-19 and post-COVID-19. Pre-COVID-19, oxygen use and length of admission were significantly higher in the BioFire group compared with the total group (16.3% vs 23.6%, p=0.05, and 3.2 days vs 4.2 days, p<0.01). However, these differences were not observed post-COVID-19 (online supplemental file 5b).

Post-COVID-19, oxygen requirement was more common in children with RSV (OR 5.1 (95% CI 2.3 to 11)) and older children (OR 1.1 (95% CI 1.0 to 1.3)), whereas this association was not significant pre-COVID-19 (OR 3.0 (95% CI 0.90 to 9.2), and OR 1.1 (95% CI 0.98 to 1.2), respectively). An additional logistic regression with the interaction of time period and RSV as a covariate was performed to exclude the possibility of this difference being caused by a power imbalance between the time periods; however, this interaction did not prove statistically significant (online supplemental file 5c). Remarkably, in the subset of young children, oxygen requirement was more common in children with rhino/enterovirus pre-COVID-19, but not post-COVID-19. By contrast, in older children, oxygen requirement was only associated with rhino/enterovirus post-COVID-19 but not pre-COVID-19 (online supplemental file 4c)

Overall, tachypnoea/dyspnoea was only significantly more common in children with RSV (OR 4.9 (95% CI 2.0 to 13)) and rhino/enterovirus (OR 2.8 (95% CI 1.6 to 5.2)) post-COVID-19, but not pre-COVID-19 pandemic (OR 3.0 (95% CI 0.90 to 14) and OR 1.4 (95% CI 0.62 to 3.2), respectively). On the other hand, tachypnoea/dyspnoea was more common in male children pre-COVID-19 (OR 0.53 (95% CI 0.30 to 0.93)), whereas this association disappeared post-COVID-19 pandemic (OR 0.75 (95% CI 0.75 to 1.3)). Notably, associations with RSV pre-COVID-19 and post-COVID-19 were only present in young children (online supplemental file 4c). An additional logistic regression analysis, incorporating the interaction between time period and rhino/enterovirus as well as time period and RSV, was performed to determine if this difference was a true effect of time and not due to a power imbalance between time periods. However, these interactions were not statistically significant (online supplemental file 5c).

### The association between seasonality and RTIs

**Figure 3:**
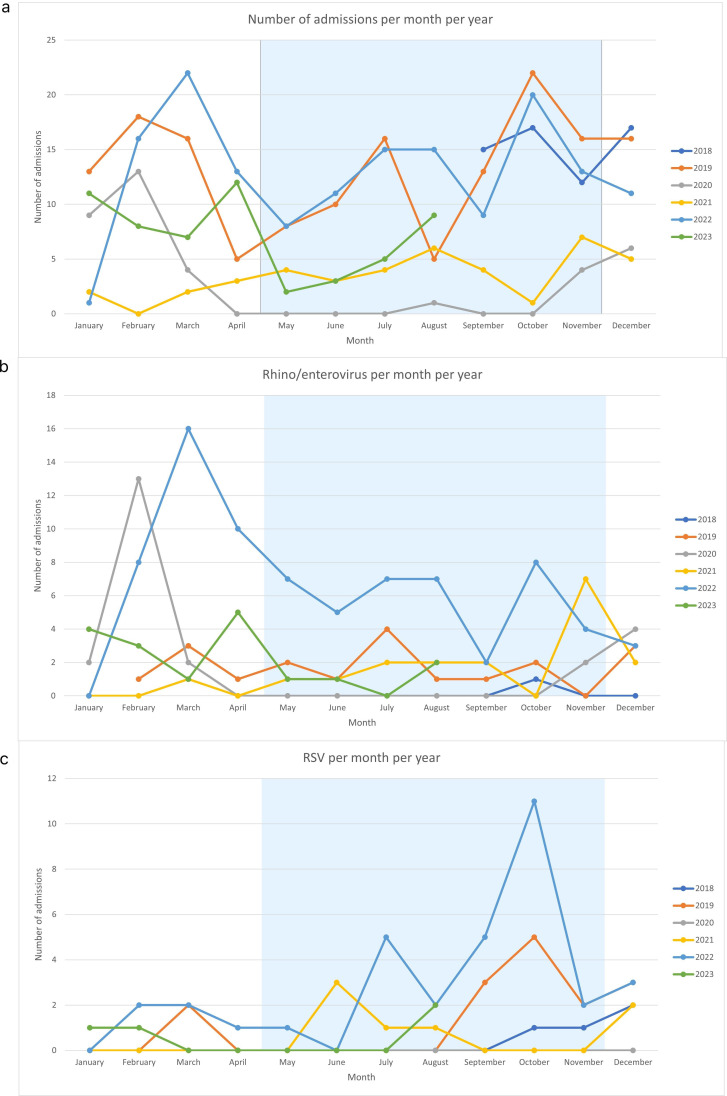
Prevalence from September 2018 to September 2023 per month of (a) all admission, (**b**) rhino/enterovirus, (**c**) RSV, with May until November marked as the rainy season. RSV, respiratory syncytial virus.

Overall, admissions were equally distributed across seasons, with 278 admissions during the rainy season and 230 during the dry season. The months with the highest cumulative numbers of admissions over the years were October (60), December (55), February (55), November (52) and March (52). In contrast, the months with the lowest numbers of admissions were May (22), June (27) and April (33). Admission numbers per month and year are shown in figure 3a.

In univariable analysis, RSV (OR 2.3, 95% CI 1.3 to 4.3, p<0.01) and parainfluenza (OR 3.0, 95% CI 1.2 to 8.4, p=0.02) were statistically significantly associated with the rainy season compared with the dry season. Conversely, rhino/enterovirus (OR 0.49, 95% CI 0.30 to 0.79, p<0.01) and influenza (OR 0.20, 95% CI 0.06 to 0.56, p<0.01) were more likely to occur during the dry season. All seasonality associations were comparable between young children and older children (online supplemental file 4d). However, when adjusting for pre-COVID-19 or post-COVID-19, none of these univariate associations remained significant.

To further assess seasonality, seasonal patterns were visualised per month (figure 3). Due to their low prevalence rates, influenza and parainfluenza were excluded from this visualisation. This showed that rhino/enterovirus did not display a consistent seasonal pattern, apart from peaks in February 2020 and March 2022. In contrast, RSV consistently peaked in June/July and September/October, both pre-COVID-19 and post-COVID-19. Hence, 84% of cases of RSV occurred between June and December. This was mainly true in young children, as 100% of cases in older children occurred between September and February.

## Discussion

Our findings shed light on the burden and severity of RTIs among children in the Caribbean. This provides valuable insights into the epidemiology and clinical characteristics of RTIs in this region, which is crucial for informing preventive strategies and healthcare management. Notably, we observed a consistently high proportion of patients diagnosed with RTIs relative to all presenting patients, with fluctuations observed in recent years. Specifically, the proportion of RTIs overall substantially decreased during the years 2020 and 2021. This decline coincided with the implementation of lockdown measures in response to the COVID-19 pandemic, suggesting potential changes in healthcare-seeking behaviour or infection transmission dynamics during periods of restricted mobility and social interaction. However, it is noteworthy that the proportion of RTI patients rebounded in 2022 and 2023, particularly among outpatients, while inpatient numbers returned to levels seen in prepandemic years. This resurgence may reflect a combination of factors, including increased testing availability, relaxed public health measures and heightened awareness of respiratory symptoms since COVID-19, and has also been observed in studies from other regions.^24 25^ Further investigation into these trends is warranted to inform targeted interventions and resource allocation for paediatric respiratory care in the Caribbean region.

We observed that non-enveloped viruses such as adenovirus and rhino/enterovirus were consistently represented post-COVID-19. These viruses have shown greater resistance to preventive measures (eg, handwashing and alcohol) employed during the COVID-19 pandemic, compared with enveloped viruses.^26^ Additionally, rhinovirus is known to be transmitted by fomites through surfaces, causing it to be persistent despite preventive COVID-19 measures.^27^ In a systematic review, adenovirus subtypes 2 and 8 were confirmed to be resistant to ethanol solution unless exposed to high concentrations for time periods longer than a minute.^28^ Ethanol solution also showed a suboptimal effect against enterovirus; however, rhinovirus seemed to be susceptible.^28^ This could explain why combined rhino/enterovirus infections did not seem to increase while a trend towards an increase in adenovirus detections was observed post-COVID-19.

Overall, the burden of RTIs in our study population was high, with RTI diagnoses accounting for 51%–67% of outpatient visits and 23%–38% of inpatient admissions outside of the COVID-19 pandemic. Inpatient percentages align with findings from other studies conducted in Latin America, which report rates between 29.6% and 38.4%.^2^ Despite this high overall burden, the disease severity, indicated by PICU admissions and mortality, was low, with rates below 2%, contrasting with higher mortality rates observed in other tropical regions of Latin America.^18^ Furthermore, only 65% of inpatients exhibited tachypnoea/dyspnoea and/or required oxygen, also indicating a lower disease severity. One possible explanation for this discrepancy could be cultural: residents of the Caribbean often seek medical attention even for mild symptoms and prefer receiving a specific diagnosis rather than adopting a wait-and-see approach, leading to high diagnosis rates. Another factor may be the island’s logistics, as severely ill patients need to be air evacuated to the nearest PICU, which may lead to precautionary admission of patients who might develop severe disease. However, despite the low mortality observed, it is important to note that our study could not investigate the long-term effects of infection, which may still pose a significant burden.

Milder diagnoses such as URTI were more common with influenza and less common with RSV and rhino/enterovirus, whereas LRTI and viral wheeze were more frequently associated with RSV and rhino/enterovirus, respectively. This suggests that RSV and rhino/enterovirus may be linked to more severe disease. This is further supported by our finding that hospitalised children with rhino/enterovirus and RSV had a higher risk of requiring oxygen or presenting with tachypnoea and/or dyspnoea at admission compared with other respiratory pathogens. These findings are supported by literature, as RSV is known to cause a higher disease burden compared with other respiratory pathogens, due to primarily affecting younger children.^29^ This was corroborated by our analysis, as RSV was more strongly associated with severe disease in young children. The association of rhino/enterovirus with severe disease contradicts the perception of it being a mild, often asymptomatic, URTI.^30 31^ However, multiple studies have shown that rhino/enterovirus can lead to severe outcomes, particularly in hospitalised cases.^31 32^ It has been suggested that rhinovirus severity is partly due to the high rate of coinfections,^33^ which is not supported by our dataset as rhino/enterovirus showed the second lowest rate of coinfection among other pathogens, although still high (32%). Additionally, rhinovirus is often detected in children with recurrent RTIs,^34^ which could explain its high prevalence in our dataset and its association with severe disease. This is further supported by the high percentage of inpatients experiencing multiple RTI admissions (15.1%) with rhino/enterovirus being more common in the children with multiple admissions than in the total cohort. This suggests that the burden of disease associated with rhino/enterovirus may be attributable to several factors besides the pathogen itself.

The COVID-19 pandemic appears to be associated with different clinical presentations of rhino/enterovirus and RSV, leading to more severe outcomes, although for rhino/enterovirus this effect was present mostly in older children, whereas for RSV it was present mostly in young children. Literature indicates that RSV infection more frequently results in severe outcomes and overall more severe disease after COVID-19,^19^ and similar trends have been observed for rhinovirus.^20^ This phenomenon may be explained by decreased exposure to pathogens and thus decreased immunity during the lockdown period, which could have a greater impact on these highly prevalent pathogens, or by the emergence of novel strains causing more severe disease.^19^ However, our findings could be influenced by a power imbalance between the time periods, as the post-COVID-19 period had roughly three times as many patients diagnosed using BioFire testing compared with the pre-COVID-19 period. While additional analyses could not exclude this possibility, the increased ORs and the shift (rather than narrowing) of the 95% CIs between pre-COVID-19 and post-COVID-19 suggest that this may still be a true effect. The shorter length of admission post-COVID-19 may be attributable to the increased BioFire testing after COVID-19 due to heightened anxiety about RTIs, leading to less selective testing and consequently diagnosing more mild cases. Thus, the cohort of tested patients post-COVID-19 would exhibit milder symptoms and be discharged sooner. This is supported by the fact that our dataset did not show a significant decrease in admission length for patients without a BioFire diagnosis. Additionally, the COVID-19 pandemic may have emphasised the need for efficient use of hospital bed capacity, encouraging faster discharge of patients.

For seasonality, RSV showed a clear and consistent pattern with multiple peaks around June/July and September/October both pre-COVID-19 and post-COVID-19. This suggests that the current timing for the RSV prophylaxis in this region is suboptimal, as administration in September or October would be too late to achieve a preventive effect. Notably, influenza showed a tendency to occur more during the dry season than the rainy season, which contradicts the literature and the WHO recommendation to vaccinate in April.^14 35^ This implies that the current vaccination schedule for influenza in September/October is already optimal. However, due to the low number of influenza detections in our dataset, it is difficult to make definitive claims about its seasonality. Remarkably, influenza seasonality coincided with tourism season in Western countries, whereas RSV seasonality did not. This can be explained by the fact that RSV affects mostly younger children who are less likely to travel, especially when sick.

Strengths of this study include the 5-year time frame of the collected data, which allowed for analysis of seasonality over multiple consecutive years. Moreover, the data captured both pre-COVID-19 and post-COVID-19 periods, enabling us to consider the COVID-19 pandemic as an effect modifier. Another strength lies in the extensive BioFire panel, which included multiple common respiratory pathogens, allowing for a comprehensive analysis comparing six major viral respiratory pathogens that contribute to disease burden and mortality in children. However, the panel lacks several relevant bacterial pathogens, including *Streptococcus pneumoniae*, *Haemophilus influenzae* and *Klebsiella pneumoniae*.

Limitations of this study include the current practice that outpatients are not eligible for pathogen testing, which restricted our analyses of this group and made it impossible to include hospitalisation as an outcome measure for different pathogens. However, we did have complete and pathogen-specific data of hospitalised patients, representing the most severely affected group. Another limitation regarding the seasonality analysis is the limited scope, as our data only represented one region in the Caribbean. Lastly, weaknesses of the study include inflation of type I error through repeated statistical testing and type II error due to limited sample size.

In conclusion, the overall burden of RTIs in children in the Caribbean part of the Kingdom of The Netherlands, as expressed in patient visits and admissions, was high, although PICU admissions and mortality were limited. Nonetheless, rhino/enterovirus and RSV are associated with more severe outcomes in this region, and the current RSV prophylaxis schedule does not optimally correspond to the observed seasonality pattern. This stresses the need to consider universal RSV prophylaxis for children and to reevaluate the timing of RSV prophylaxis administration. These findings should be confirmed using a larger cohort covering multiple regions of the Caribbean.

## Supplementary material

10.1136/bmjopen-2025-104991 online supplemental file 1

## Data Availability

Data are available on reasonable request.
