# Supplementary material for: Association between common respiratory pathogens and disease severity, and pathogen-specific seasonality in the Caribbean pre-COVID-19 and post-COVID-19: a retrospective study
Source: BMJ Open. 2026 Jan 7;16(1):e104991. doi: 10.1136/bmjopen-2025-104991 (PMC12781987; doi:10.1136/bmjopen-2025-104991)

**Supplementary 1**

ICD-10 codes

J029 Acute pharyngitis, unspecified

J028 Acute pharyngitis due to other specified organism

J0380 Streptococcal tonsillitis

J0390 Acute tonsillitis

J0391 Acute recurrent tonsillitis

J040 Acute Laryngitis

J042 Acute laryngotracheitis

J050 Acute obstructive laryngitis and epiglottis

J069 Acute upper respiratory tract infection, unspecified

J180 Bronchopneumonia, unspecified

J181 Lobar pneumonia, unspecified

J189 Pneumonia, unspecified

J09X2 Influenza Due to Identified Novel Influenza A Virus

J09X3 Influenza due to identified novel influenza A virus with gastrointestinal

manifestations

J101 Influenza due to other identified influenza virus with other respiratory

manifestations

J111 Influenza due to unidentified influenza virus with other respiratory

manifestations

J123 Human metapneumovirus pneumonia

J1281 Pneumonia due to SARS-associated coronavirus

J1282 Pneumonia due to coronavirus disease 2019

J1289 Viral pneumonia, unspecified

J129 Viral pneumonia, unspecified

J151 Pneumonia due to Pseudomonas

J157 Pneumonia due to Mycoplasma pneumoniae

J159 Bacterial pneumonia, unspecified.

J168 Pneumonia due to other specified infectious organisms

J204 Acute bronchitis due to parainfluenza virus

J206 Acute bronchitis due to rhinovirus

J208 Acute bronchitis due to other specified organism

J209 Acute bronchitis, unspecified

J210 Acute bronchiolitis due to respiratory syncytial virus

J211 Acute bronchiolitis due to HMPV

J218 Acute bronchiolitis due to other specified organism

J219 Acute bronchiolitis, unspecified

J22 Unspecified ALRI

J9801 Acute bronchospasm

R05 Cough

R060 Dyspnea

R061 Stridor

R062 Viral wheeze

**Supplementary 2**

Case Report Form

| **Variable:** | **Answer:** |
| --- | --- |
| Date of birth |  |
| Age of the child (years) |  |
| Gender | ☐ Male  ☐ Female |
| Admitted in the hospital | ☐ Yes - Inpatient  ☐ No - Outpatient |
| Date of admission |  |
| Date of discharge |  |
| Length of admission (days) |  |
| PICU admission | ☐ Yes  ☐ No |
| Readmission | ☐ Yes  ☐ No |
| Reason of admission | ☐ Oxygen  ☐ IV treatment  ☐ Intake  ☐ Monitoring  ☐ Nebulizer  ☐ Social  ☐ Missing |
| Time of presentation | ☐ 07:30 – 13:00  ☐ 13:00 – 18:00  ☐ 18:00 – 07:30 |
| Route of admission | ☐ Outpatient dept  ☐ Emergency dept  ☐ Unknown |
| Has the patient undergone biofire panel? | ☐ Yes  ☐ No |
| How many pathogens were detected with biofire panel? |  |
| Biofire result | ☐ Adenovirus  ☐ Coronavirus 229E  ☐ Coronavirus HKU1  ☐ Coronavirus NL63  ☐ Coronavirus OC43  ☐ SARS-CoV-2  ☐ Human Metapneumovirus  ☐ Human Rhinovirus/Enterovirus  ☐ Influenza A virus  ☐ Influenza A virus A/H1  ☐ Influenza A virus A/H3  ☐ Influenza A virus A/H1-2009  ☐ Influenza B virus  ☐ Parainfluenza virus 1  ☐ Parainfluenza virus 2  ☐ Parainfluenza virus 3  ☐ Parainfluenza virus 4  ☐ Respiratory syncytial virus  ☐ Bordetella parapertussis  ☐ Bordetella pertussis  ☐ Chlamydia pneumoniae  ☐ Mycoplasma pneumoniae  ☐ No infections |
| Symptoms at admission | ☐ Fever  ☐ Cough  ☐ Wheezing  ☐ Abnormal auscultation  ☐ Dyspnea  ☐ Tachypnea  ☐ Upper respiratory symptoms  ☐ Gastritis (vomiting or diarrhea)  ☐ None of the mentioned  ☐ Febrile seizure |
| Oxygen requirement | ☐ Yes  ☐ No |
| Ill appearance | ☐ Yes  ☐ No  ☐ Missing |
| Duration of fever (days) |  |
| Retractions | ☐ Yes  ☐ No  ☐ Missing |
| Capillary refill time (second) |  |
| Temperature (Celsius) |  |
| Oxygen saturation at room air (%) |  |
| Heart rate (beats per minute) |  |
| Respiratory rate (breaths per minute) |  |
| Level of consciousness | ☐ Alert  ☐ Verbal  ☐ Pain  ☐ Unresponsive  ☐ Missing |
| X-ray admission | ☐ Yes  ☐ No |
| Was there pneumonia present? If yes, please indicate what type. |  |
| Full radiology report |  |
| Were any lab tests done? | ☐ Yes  ☐ No |
| Enter lab results  CRP  Hb  Leukocytes  Thrombocytes  Neutrophil  Lymphocyte  Monocyte  Eosinophil  Basophil  BSE  Other, specify  Other, specify |  |
| Diagnosis | ☐ Pneumonia  ☐ Bronchiolitis  ☐ Asthma exacerbation  ☐ Viral wheeze  ☐ AURI  ☐ LRTI  ☐ Other |
| Antibiotics prescription | ☐ Yes  ☐ No |
| Length of antibiotic use (days) |  |
| Which antibiotic was prescribed | ☐ Amoxicillin  ☐ Augmentin  ☐ Azithromycin  ☐ Other |
| Please specify which other antibiotic was prescribed |  |
| Treating physician |  |
| Was the patient treated with antibiotics before admission | ☐ Yes  ☐ No  ☐ Missing |
| Patient history | ☐ Asthma  ☐ Prematurity  ☐ Allergies  ☐ Sickle cell disease  ☐ Down syndrome  ☐ Cystic fibrosis  ☐ Chronic constipation/GI surgery  ☐ Congenital heart disease  ☐ None |
| Cardiovasulcar disease/congenital heart disease? | ☐ Yes  ☐ No  ☐ Missing |
| Asthma | ☐ Yes  ☐ No  ☐ Missing |
| Other chronic pulmonary disease (not asthma) | ☐ Yes  ☐ No  ☐ Missing |
| Immune deficiency | ☐ Yes  ☐ No  ☐ Missing |
| Malignancy | ☐ Yes  ☐ No  ☐ Missing |
| Sickle cell disease | ☐ Yes  ☐ No  ☐ Missing |
| Other haematologic disease (not sickle cell) | ☐ Yes  ☐ No  ☐ Missing |
| Genetic defects | ☐ Yes  ☐ No  ☐ Missing |
| Prematurity | ☐ Yes  ☐ No  ☐ Missing |
| Other comorbidity | ☐ Yes  ☐ No |

**Supplementary 3**

Overview of outpatients, RTI diagnoses and hospital admissions over the time period of September 1, 2018, until September 1, 2023.


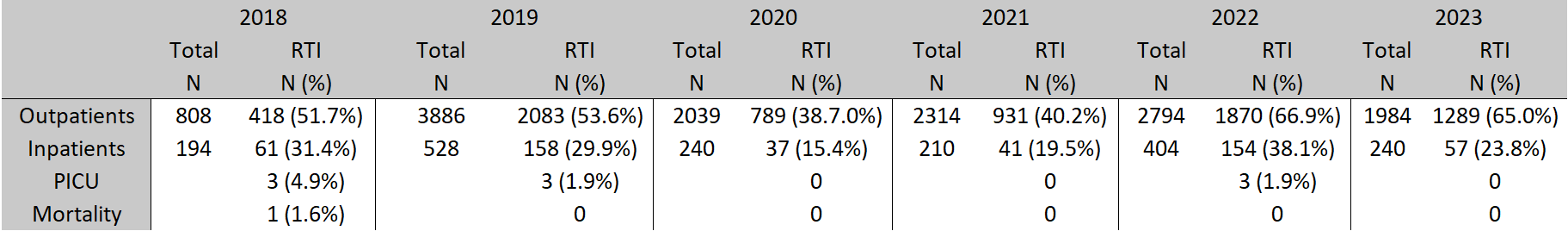


**Supplementary 4**

**a) Pathogen frequencies stratified by age**

|  | Total | | Pre-Covid-19 | | Post-Covid-19 | |
| --- | --- | --- | --- | --- | --- | --- |
| **Pathogen** | Young Children (≤2 years) | Older Children (>2 years) | Young Children (≤2 years) | Older Children (>2 years) | Young Children (≤2 years) | Older Children (>2 years) |
| *Adenovirus - N (%)* | 22 (13.3%) | 8 (9.6%) | 4 (9.3%) | 0 (0.0%) | 18 (14.6%) | 8 (12.7%) |
| *SARS-CoV-2 - N (%)* | 11 (6.6%) | 1 (1.2%) | 0 (0.0% | 0 (0.0%) | 11 (8.9%) | 1 (1.6%) |
| *HMPV - N (%)* | 9 (5.4%) | 7 (8.4%) | 2 (4.7%) | 1 (5.0%) | 7 (5.7%) | 6 (9.5%) |
| *RSV - N (%)* | 56 (33.7%) | 8 (9.6%) | 17 (39.5%) | 2 (10.0%) | 39 (31.7%) | 6 (9.5%) |
| *Influenza - N (%)* | 10 (6.0%) | 9 (10.8%) | 2 (4.7%) | 2 (10.0%) | 8 (6.5%) | 7 (11.1%) |
| *Parainfluenza - N (%)* | 22 (13.3%) | 4 (4.8%) | 8 (18.6%) | 1 (5.0%) | 14 (11.4%) | 3 (4.8%) |
| *Mycoplasma pneumoniae - N (%)* | 0 (0.0%) | 2 (2.4%) | 0 (0.0%) | 1 (5.0%) | 0 (0.0%) | 1 (1.6%) |
| *Rhino/enterovirus - N (%)* | 93 (56.0%) | 62 (74.7%) | 23 (53.5%) | 14 (70.0%) | 70 (56.9%) | 48 (76.2%) |

**b) Associations between pathogens and severity outcomes stratified by age**

**
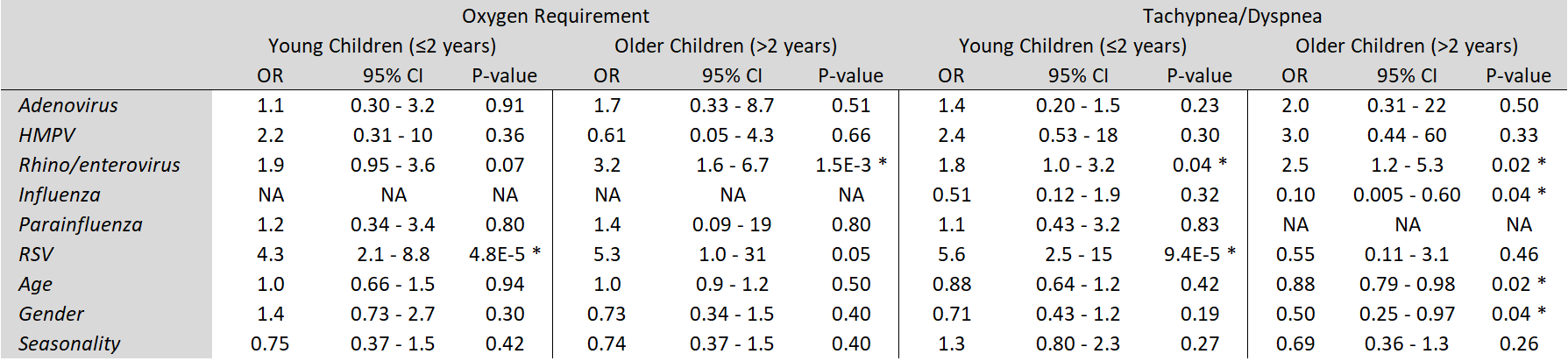
**

**c) Associations between pathogens and severity outcomes pre- and post-COVID-19 stratified by age**

**
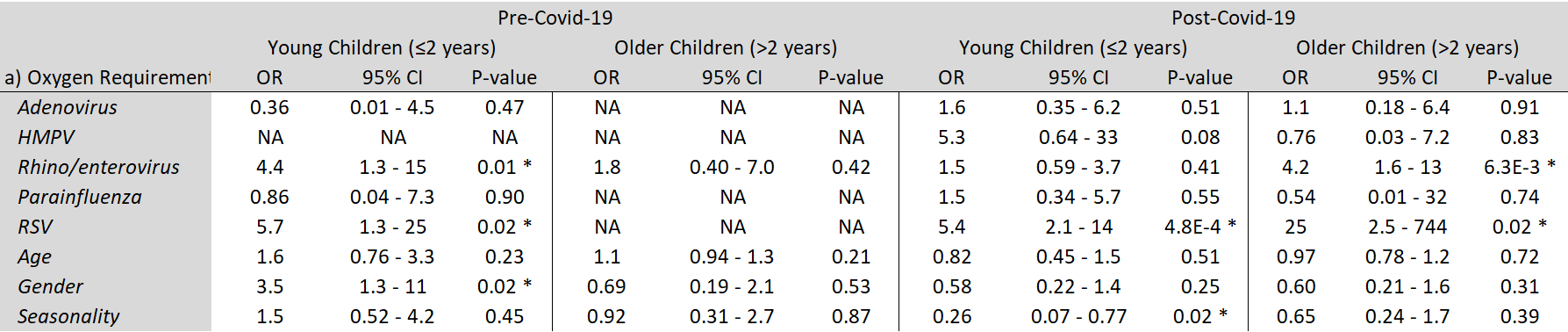
**

**
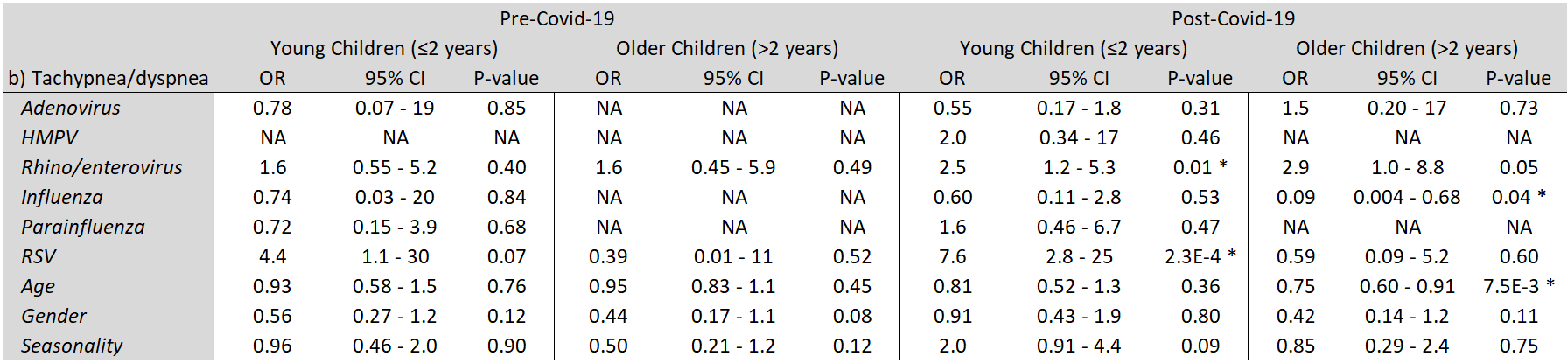
**

**d) Associations between pathogens and seasonality stratified by age**

|  | Young Children (≤2 years) | | Older Children (>2 years) | |
| --- | --- | --- | --- | --- |
| **Pathogen** | Rainy Season (OR) | P-value | Rainy Season (OR) | P-value |
| *Rhino/enterovirus* | 0.6 | 0.06 | 0.5 | 0.1 |
| *RSV* | 1.9 | 0.06 | 1.6 | 0.5 |
| *Influenza* | 0.2 | 0.04 | 0.2 | 0.1 |
| *Parainfluenza* | 3.0 | 0.06 | 1.6 | 0.7 |

**Supplementary 5**

1. **Comparison BioFire group pre- and post-COVID-19**


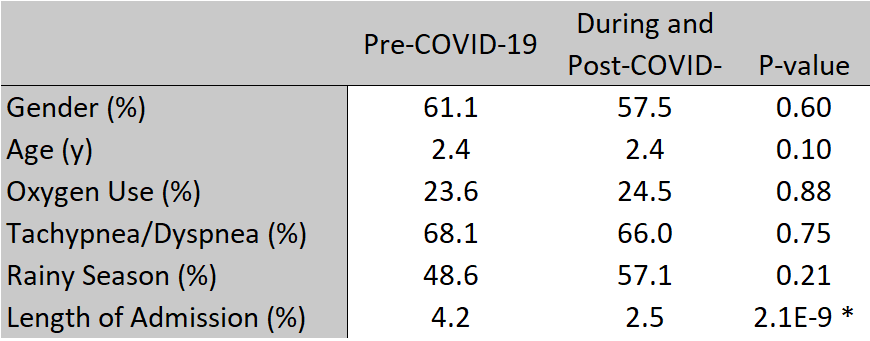


1. **Comparison BioFire group to total group, pre- and post-COVID-19**

**
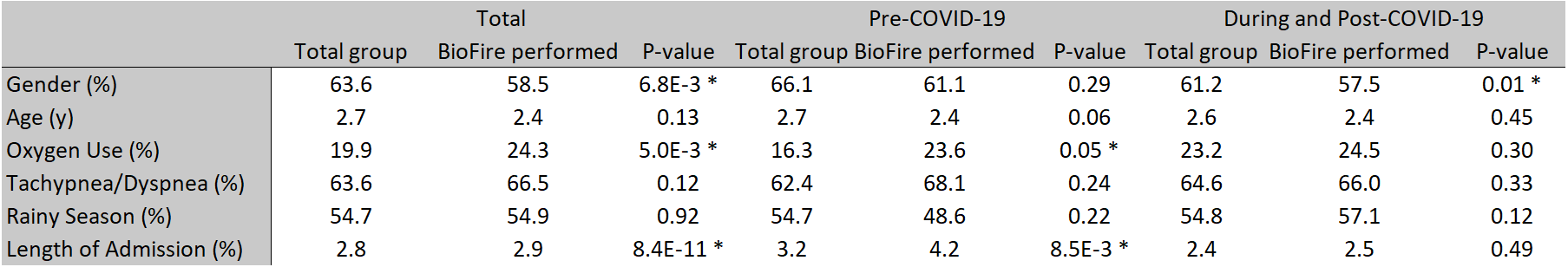
**

1. **Associations between pathogens and disease severity pre- and post-COVID-19**


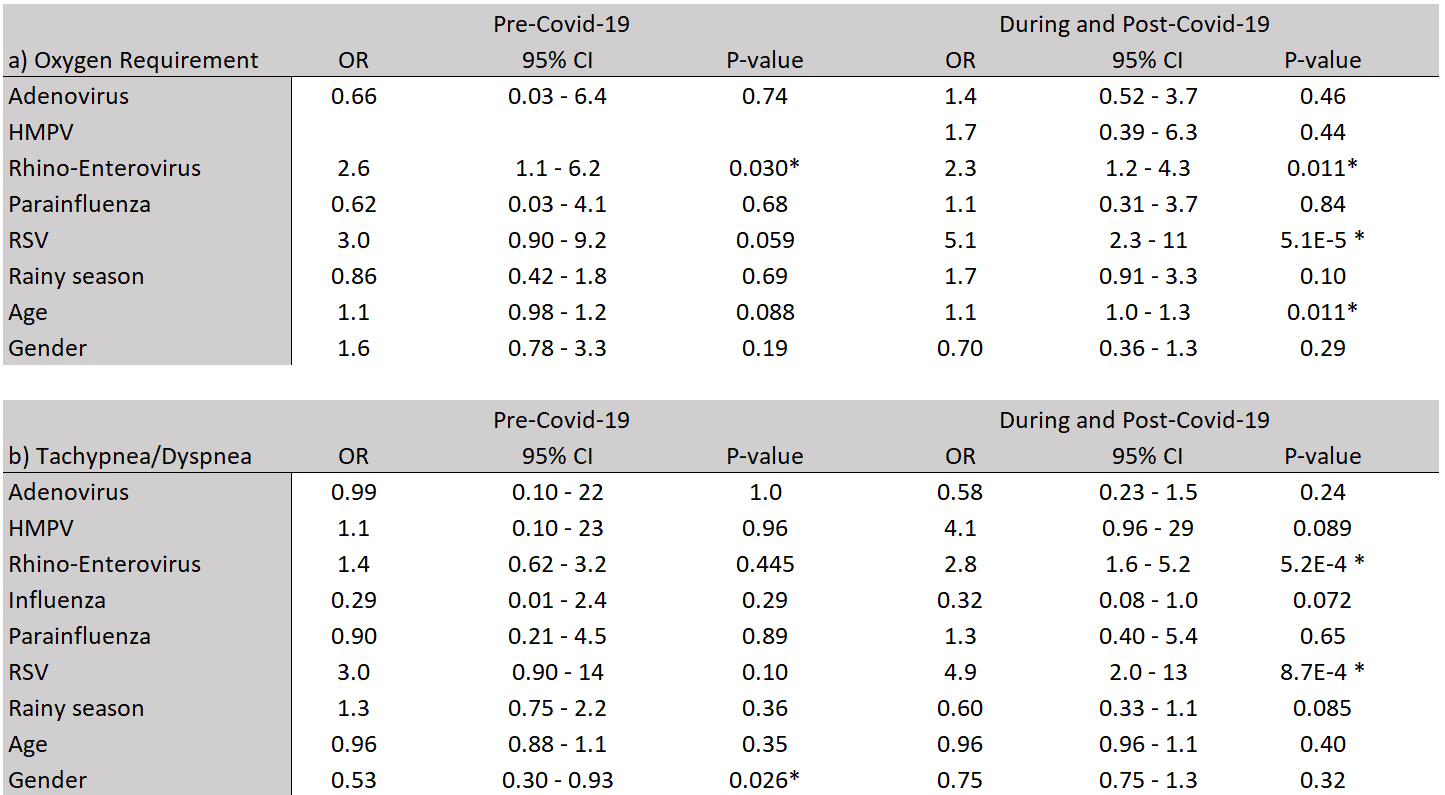

Supplement: online supplemental file 1 [file bmjopen-16-1-s001.docx]
