# Supplementary material for: Association between common respiratory pathogens and disease severity, and pathogen-specific seasonality in the Caribbean pre-COVID-19 and post-COVID-19: a retrospective study
Source: BMJ Open. 2026 Jan 7;16(1):e104991. doi: 10.1136/bmjopen-2025-104991 (PMC12781987; doi:10.1136/bmjopen-2025-104991)
Supplement: Supplementary file 2 [file bmjopen-2025-104991.reviewer_comments.pdf]

PEER REVIEW HISTORY

BMJ Open publishes all reviews undertaken for accepted manuscripts. Reviewers are asked to complete a checklist review form and are provided with free text boxes to elaborate on their assessment. These free text comments are reproduced below.

ARTICLE DETAILS

Title (Provisional)

Association between common pathogens and disease severity, and pathogen specific seasonality in the Caribbean pre- and post-COVID-19: a retrospective study

Authors

Engels, Sam; Tilanus, Martijn; Juliet, Juldany; Euson, Marquita; Stastny, Nika; Maria, Charlene; Baboe-Kalpoe, Sharda; Baboe , Fazal; Fleming, Chérina KA; Carty-Fleming, Sherryl; Holaman, Shanna; Baan, Kaylee; Holiday, Felix; Van den Borne, Sebastiaan CF; Plantinga, Rutger F; Wong Chung, Dick; van de Maat, Josephine; de Jonge, Marien I; Steingrover, Radjin; Verhagen, Lilly M.

VERSION 1 - REVIEW

**Reviewer**	**1**
**Name**	**Izu, Alane**
**Affiliation**	**University of the Witwatersrand Johannesburg**
**Date**	**12-Aug-2025**
**COI**	**None**

This manuscript describes results from an observational study aimed to estimate disease burder of RTI and LRTI, as well as aetiologies of RTI, LRTI and URTI. It also describes the seasonality or RSV which is important in establishing public health policy.

The objective can be empahasized better and stratified analysis by in-patient/out-patient and age should be performed.

Page 3:

Line 9: Referring to "assess disease burden" - What disease? RSV? Influenza? RTI?

Line 29: This sentence doesn't speak to what is already known.

Line 26: "RTI are a major contributor to disease burden" - doesn't make sense. Disease burden refers to the impact of a disease. RTI is a major contributor to the impact of disease.

Line 34 and 36: These sentences describe the same point. Illustrating the seasonality of RSV and Influenza is the same as identifying pathogen seasonality. Remove the word "finally."

Line 34: change to "Identifying pathogens associated with disease severity and describing pathogen specific seasonality.

Line 36: The Caribbean is part of North America, has data from Caribbean countries been analysed with data from South America?

Line 40: "The burden of respiratory disease burden" - remove "burden" after disease.

Please cite GBD 2021.

Page 4:

Line 6 - "Specifically, lower respiratory tract infections..."

Can the first paragraph be separated into smaller more focused paragraphs? The focus begins with RTI/LRTI, then speaks to RSV and other pathogens, then speaks to RSV treatment and then seasonality of RSV in different regions.

Line 35-38 - there should be a few references that speak to this - there may be some that speak to the influenza seasonality changes during COVID and how travel may have been a contributing factor.

Line 46 - the last two sentences of the introduction speak clearly to the objectives. Unfortunately, this doesnt come out in the abstract or the title. The title and abstract should be worded to reflect the objectives of the study which are stated clearly in these two sentences.

Page 5:

Line 26 - how many had missing discharge information?

Line 12 - This sentence can go in the Ethics subsection

Line 54 - Which descriptive statistics?

Line 56/59 - What does "pathogens" refer to? Is it the association between incidence of identification of a specific pathogen (i.e a positive lab result for that pathogen?) and RTI/LRTI and clinical severity?

Please include a brief description of the diagnostic tool - is it used to identify pathogens from a sample?

Page 7

Line 4 - Wilcoxon rank-sum test and Chi-square test are tools for inference and not descriptive. Descriptive statistics are statistics which describe the data.

Line 10 - Which assumptions?

Line 25 - Does not need to begin on a new line

Line 36 - Did not everyone had BioFire? Did the authors do a sensitivity analysis to impute results for admissions that did not have BioFire? Also, in the methods section, line 16 states

"missingness was negligible in the dataset" which contradicts that BioFire wasn't available for all patients.

Page 9

Line 36-41 - It makes sense to differentiate between in-patient and out-patient. Can the authors stratify the analysis by in-patient/out-patient?

Line 43 - would be informative to have a stratified analysis by age group as well. Which pathogens were common among which age groups?

Pathogens, seasonality and clinical outcomes should be an age-stratified analysis.

Figure 2: Can an additional attribute be used to distinguish between pre and post? (Shading?)

If the aim is to describe the seasonality of RSV and influenza to determine if the current strategy of preventative measures is optimal, then rather just state that a specific objective is to describe the seasonality of RSV and influenza. Then describe the seasonality of RSV and influenza in the last section of the results. This should be done by age group (<3, 3-12m, 1-2y, 2-5yr 5-9,>=10)

Line 37 - more descriptive statistics can be presented. XX% of all RSV happens between XX and XX. Alternatively, RSV seasonality has been defined as the time the number of cases is above a threshold until when it is lower than a threshold. Please use this type of definition and describe the seasonality of RSV and influenza.

Page 9

Line 47 - how does this compare to published studies from different regions?

Page 11

Line 3 - is this in in-patient or in out-patient study populations? Or overall?

Page 10

Line 27-38 - Its well known that RSV is associated with LRTIs. If authors want to emphasise this, perhaps use 1-2 sentences as there are more important results to discuss (seasonality, age-stratified analysis, etc).

Page 11

Line 7 - this is important and perhaps should be mentioned earlier in the discussion.

There is no mention of tourism seasonality in the discussion. Please include this.

Reviewer 2

**Name**	**Weatherall, Mark**
**Affiliation**	**University of Otago Wellington**
**Date**	**01-Sep-2025**
**COI**	**None**

The manuscript reports a retrospective cohort study of children admitted to hospital in a single health jurisdiction with respiratory tract infection.

A weakness of the manuscript is the absence of a specific listed set of research aims. Based on the reporting of the results these research aims were likely: Identifying different characteristics of children admitted to hospital with a respiratory tract infection before and during or after COVID-19 became prevalent (described in Table 1), describing associations between different respiratory infection syndromes and disease and participant characteristics (left-hand columns of Table 2), describing associations between respiratory infection severity indicators and disease and participant characteristics (right-hand columns of Table 2), describe disease associations with seasonality (page 9 lines 25-40 and associated plots).

In the statistical methods although logistic and linear regression are specified as methods it was difficult to see where linear regression was used as most results are reported as odds ratios.

For Table 1 it might be better to separate it into two sections, one reporting scale variables and one reporting categorical variables, so that the two sections could have common column headers and it would be better to have a more fulsome description of the summary data for the scale variables e.g. include mean and SD. For this table there should be reporting of appropriate measures of association with a confidence interval and not just a P; and additionally, it is poor statistical practice to report boundary P values e.g. P<0.01. Reporting of P-values should be to appropriate precision as per the journal requirements. For some of the categorical variable associations it was unclear how this was handled e.g. if there were a set of mutually exclusive categories, then there should be an overall P-value for any evidence of a difference in proportions e.g. the 'Diagnosis' was likely mutually exclusive so there should be one P-value for association and odds ratios could be reported with reference to a baseline or reference level. It was also unclear how the scale variables were compared e.g. t-tests versus rank-based tests.

For Table 2 it was unclear who the variables were chosen for the explanatory side of the logistic regression as these different slightly between the different response variables. This table would be better if there were numeric descriptors e.g. numerators/denominators and appropriate scale variable data descriptors per predictor in relation to response variable. Again, boundary P values are inappropriate.

In the weaknesses should be included Type I error inflation through repeated statistical testing and Type II error for detecting important associations limited by sample size.

VERSION 1 - AUTHOR RESPONSE

Reviewer: 1

Dr. Alane Izu, University of the Witwatersrand Johannesburg

Comments to the Author:

This manuscript describes results from an observational study aimed to estimate disease burder of RTI and LRTI, as well as aetiologies of RTI, LRTI and URTI. It also describes the seasonality or RSV which is important in establishing public health policy.

The objective can be empahasized better and stratified analysis by in-patient/out-patient and age should be performed.

We thank Dr Izu for the thoughtful and constructive review, and for the positive assessment of the manuscript's relevance and contribution to public-health policy.

Page 3:

Line 9: Referring to "assess disease burden" - What disease? RSV? Influenza? RTI?

Response: We clarified that the term "disease burden" specifically refers to the overall respiratory tract infection (RTI) disease burden. The text was amended accordingly to avoid any ambiguity.

Line 26: "RTI are a major contributor to disease burden" - doesn't make sense. Disease burden refers to the impact of a disease. RTI is a major contributor to the impact of disease.

Response: We have removed this paragraph at the Editor's request.

Line 29: This sentence doesn't speak to what is already known.

Response: We have removed this paragraph at the Editor's request.

Line 34 and 36: These sentences describe the same point. Illustrating the seasonality of RSV and Influenza is the same as identifying pathogen seasonality. Remove the word "finally."

Response: We have removed this paragraph at the Editor’s request.

Line 34: change to "Identifying pathogens associated with disease severity and describing pathogen specific seasonality.

Response: We have removed this paragraph at the Editor’s request.

Line 36: The Caribbean is part of North America, has data from Caribbean countries been analysed with data from South America?

Response: We have removed this paragraph at the Editor’s request. The original wording reflected the categorisation used in GBD 2019 (GBD 2019 LRI Collaborators, *Age–sex differences in the global burden of lower respiratory infections and risk factors, 1990–2019: results from the Global Burden of Disease Study 2019*, The Lancet, 2022), which groups the Caribbean with Latin America, primarily South America. To ensure clarity and to align with the reviewer’s suggestion, we have changed the wording to “Latin America” for clarity in the revised manuscript.

Line 40: "The burden of respiratory disease burden" - remove "burden" after disease.

Response: We have removed this paragraph at the Editor’s request.

Please cite GBD 2021.

Response: Response: We have removed this paragraph at the Editor’s request. However, we now cited GBD 2021 in the Introduction.

<u>Page 4:</u>
Line 6 - "Specifically, lower respiratory tract infections..."
Can the first paragraph be separated into smaller more focused paragraphs? The focus begins with RTI/LRTI, then speaks to RSV and other pathogens, then speaks to RSV treatment and then seasonality of RSV in different regions.

Response: Thank you for this helpful suggestion. We have restructured the opening to improve focus and readability. The first paragraph now addresses RTIs and LRTIs in general. The second paragraph focuses on RSV. We introduced the paragraph break at line 6, as recommended by the reviewer.

Line 35-38 - there should be a few references that speak to this - there may be some that speak to the influenza seasonality changes during COVID and how travel may have been a contributing factor.

Response: We have added the following references to this sentence:

Quintero-Salgado E, Briseno-Ramírez J, Vega-Cornejo G, Damian-Negrete R, Rosales-Chavez G, De Arcos-Jiménez JC. Seasonal Shifts in Influenza, Respiratory Syncytial Virus, and Other Respiratory Viruses After the COVID-19 Pandemic: An Eight-Year Retrospective Study in Jalisco, Mexico. Viruses. 2024 Dec 8;16(12):1892.

Chen, Z., Tsui, J.LH., Cai, J. et al. Disruption of seasonal influenza circulation and evolution during the 2009 H1N1 and COVID-19 pandemics in Southeastern Asia. Nat Commun 16, 475 (2025). https://doi.org/10.1038/s41467-025-55840-y

Line 46 - the last two sentences of the introduction speak clearly to the objectives. Unfortunately, this doesnt come out in the abstract or the title. The title and abstract should be worded to reflect the objectives of the study which are stated clearly in these two sentences.

Response: We changed the last sentence of the abstract to "We aimed to investigate pathogens associated with disease burden and pathogen specific seasonality in the Caribbean in the context of COVID-19 to achieve optimal timing of preventive measures." We changed the title to "Association between common pathogens and disease severity, and pathogen specific seasonality in the Caribbean pre- and post-COVID-19: a retrospective study." to reflect the study aims more clearly.

<u>Page 5:</u>
Line 12 - This sentence can go in the Ethics subsection

Response: We moved the sentence to the Ethics subsection.

Line 26 - how many had missing discharge information?

Response: The number of cases with missing discharge information was very minimal, as discharge details are routinely documented in standardized discharge letters at our hospital. Consequently, only a negligible number of cases were excluded for this reason.

Line 54 - Which descriptive statistics?

Response: We used the mean and standard deviation for normally distributed variables and the median and range for non-normally distributed variables. For pathogens specifically we used percentages. We have added this in the text to avoid ambiguity.

Line 56/59 - What does "pathogens" refer to? Is it the association between incidence of identification of a specific pathogen (i.e a positive lab result for that pathogen?) and RTI/LRTI and clinical severity?
Please include a brief description of the diagnostic tool - is it used to identify pathogens from a sample?

Response: With "pathogens," we refer to pathogens detected using the *BioFire®* Respiratory Panel diagnostic tool. We have now clarified this in the Methods section ("Study population and definitions") and added a brief description of the BioFire®, which identifies respiratory pathogens directly from patient samples using multiplex PCR. To address the reviewer's question: yes, the analyses describe the association between the identification (positive detection) of specific pathogens by the BioFire® and RTI/LRTI diagnoses as well as clinical severity.

Page 7
Line 4 - Wilcoxon rank-sum test and Chi-square test are tools for inference and not descriptive. Descriptive statistics are statistics which describe the data.

Response: We fully agree with the reviewer's comment, and we changed the wording to "Additional inferential statistics" instead.

Line 10 - Which assumptions?

Response: We checked for multicollinearity using a VIF score and we checked the distribution of residuals. We added this to the methods for clarity.

Line 25 - Does not need to begin on a new line

Response: We removed the enter, so that the sentence does not start on a new line.

Line 36 - Did not everyone had BioFire? Did the authors do a sensitivity analysis to impute results for admissions that did not have BioFire? Also, in the methods section, line 16 states "missingness was negligible in the dataset" which contradicts that BioFire wasn't available for all patients.

Response: Thank you for this thoughtful comment. BioFire testing was performed in 55.9% of admitted children. For pathogen-specific analyses, we restricted the dataset to those with BioFire results, since the main predictor would otherwise be entirely missing. Within this analytic subset, missingness of outcome measures was indeed negligible. We recognise that our wording on subset use and missingness could be clearer. We have therefore revised the Methods to state explicitly that pathogen-association analyses were restricted to admissions with BioFire results, and to specify that “missingness of outcome measures was negligible in the dataset”. In addition, we conducted sensitivity analyses to assess generalizability by comparing outcome measures between admissions with and without BioFire testing. These analyses are reported in the Results section under “Pre- and post-COVID-19 comparison of clinical outcomes”. Pre-COVID-19, oxygen use and length of stay were significantly higher in the BioFire group than in the total cohort. These differences were not observed post-COVID-19 (Supplementary Table 5b in the revised manuscript). This suggests some pre-pandemic selection of sicker patients for testing. However, as our pathogen-association analyses were restricted to the BioFire subset, the internal validity of those analyses is preserved, and we have clarified this approach in the Methods of the revised manuscript.

Page 9
Line 36-41 - It makes sense to differentiate between in-patient and out-patient. Can the authors stratify the analysis by in-patient/out-patient?

Response: All analyses beyond the first paragraph of the results are only conducted in inpatients, as we did not have detailed data on the outpatients. Therefore, we cannot stratify the data this way. However, we now have more clearly emphasized that only inpatients were included by changing the line “Further analyses focused on the inpatient population…” to “Further analyses only included the inpatient population…”

Line 37 - more descriptive statistics can be presented. XX% of all RSV happens between XX and XX. Alternatively, RSV seasonality has been defined as the time the number of cases is above a threshold until when it is lower than a threshold. Please use this type of definition and describe the seasonality of RSV and influenza.

Response: We added additional descriptive statistics, describing the percentage of RSV cases happening between certain months. The threshold we used for seasonality was the 75th percentile over the entire study period for the specific pathogen, which was 2 cases per month for RSV. Therefore, each month with a number of cases higher than 2 is considered a peak, and the period in which all peaks occurred during the study period is considered the seasonality. We described this more clearly in the Methods section of our revised manuscript to avoid unclarity.

Line 43 - would be informative to have a stratified analysis by age group as well. Which pathogens were common among which age groups?
Pathogens, seasonality and clinical outcomes should be an age-stratified analysis.

Response: We have added subgroup analyses stratified by age group as suggested by the reviewer. We have added this in the following sentences:

In the Methods (page 5, line 13-14):

Additionally, we performed subset analyses on all outcomes for young children (aged ≤2 years) and older children (aged >2 years).

In the Results (page 5, line 39-46):

LRTI was the most common diagnosis in young children (45·3%), followed by URTI and viral wheeze (29·3% and 25·7% respectively), with no cases of asthma exacerbation as by definition this could only occur in older children. On the other hand, older children most commonly experienced viral wheeze (32·0%), followed by URTI and LRTI (25·9% and 25·4%) and finally asthma exacerbation (16·2%). In young children, the most common pathogen was rhino/enterovirus, constituting 56·0% of all detected pathogens, followed by RSV (33·7%), adenovirus (13·3%) and parainfluenza (13·3%). In older children, however, the two most common pathogens were rhino/enterovirus (74·7%) and influenza (10·8%) (Supplementary 4a).

(page 6, line 6-7)

Notably, these changes mostly reflect trends in young children, whereas in older children, only adenovirus showed an increase from 0·0% to 12·7% whereas other pathogens remained stable.

(page 7, line 14-20)

Rhino/enterovirus, RSV and age showed significant positive associations with the need for oxygen treatment during admission, with ORs of 2·3 (95% CI 1·4 – 3·8), 4·2 (95% CI 2·2 - 7·7), and 1·1 (95% CI 1·0 - 1·2, p=2.4E-3), respectively (Table 2d), which was similar in young and older children, although the association with rhino/enterovirus was stronger in older children (Supplementary 4b). Regarding tachypnea and/or dyspnea, rhino/enterovirus and RSV showed statistically significant positive associations, with ORs of 2·0 (95% CI 1·3 – 3·1) and 3·6 (95% CI 1·8 – 8·0), respectively. However, this association with RSV was only present in young children (Supplementary 4b).

(page 8, line 25-28)

Remarkably, in the subset of young children, oxygen requirement was more common in children with rhino/enterovirus pre-COVID-19, but not post-COVID-19. By contrast, in older children oxygen requirement was only associated with rhino/enterovirus post-COVID-19 but not pre-COVID-19 (Supplementary 4c)
Notably, associations with RSV pre- and post-COVID-19 were only present in young children (Supplementary 4c).

(page 9, line 4-5)

All seasonality associations were comparable between young children and older children (Supplementary 4d).

(page 9, line 11-12)

Hence, 84% of cases of RSV occurred between June and December. This was mainly true in young children, as 100% of cases in older children occurred between September and February.

In the Discussion (page 10, line 7-8):

This was corroborated by our analysis, as RSV was more strongly associated with severe disease in young children.

(page 10, line 19-20)

The COVID-19 pandemic appears to have influenced the clinical presentations of rhino/enterovirus and RSV, leading to more severe outcomes, although for rhino/enterovirus this effect was present mostly in older children, whereas for RSV it was present mostly in young children

Figure 2: Can an additional attribute be used to distinguish between pre and post?

(Shading?)

Response: We changed the colors of the post-COVID-19 boxplots in order to improve the interpretability of the figure.

If the aim is to describe the seasonality of RSV and influenza to determine if the current strategy of preventative measures is optimal, then rather just state that a specific objective is to describe the seasonality of RSV and influenza. Then describe the seasonality of RSV and influenza in the last section of the results. This should be done by age group (<3, 3-12m, 1-2y, 2-5yr 5-9,>=10)

Response: Our overarching aim was not to describe seasonality of RSV and influenza to determine whether the current strategy of preventative measures is optimal, but rather to describe seasonality of the pathogens that are associated with paediatric disease burden in order to determine whether preventive strategies for those pathogens (if available) are optimal. This is now described in the Introduction of our revised manuscript. In line with this recommendation, we have now split the univariable seasonality analysis stratified by age group. These comparisons did not show material differences between age strata, and this has been clarified in the revised manuscript.

Page 9

Line 47 - how does this compare to published studies from different regions?

Response: These findings are similar to other published studies from different regions. We added the following additional references for this sentence:

Alexandre Sabaté-Elabbadi, Lucie Brolon, Christian Brun-Buisson, Didier Guillemot, Muriel Fartoukh, Laurence Watier, Trends in hospitalisations for lower respiratory infections after the COVID-19 pandemic in France, Journal of Infection, 2024;89(5)

Izu A, Nunes MC, Solomon F, Baillie V, Serafin N, Verwey C, Moore DP, Laubscher M, Ncube M, Olwagen C, Dangor Z, Madhi SA. All-cause and pathogen-specific lower respiratory tract infection hospital admissions in children younger than 5 years during the COVID-19 pandemic (2020-22) compared with the pre-pandemic period (2015-19) in South Africa: an observational study. Lancet Infect Dis. 2023 Sep;23(9):1031-1041.

Page 10

Line 27-38 - Its well known that RSV is associated with LRTIs. If authors want to emphasise this, perhaps use 1-2 sentences as there are more important results to discuss (seasonality, age-stratified analysis, etc).

Response: We acknowledge that this is indeed well known from literature. We have shortened the paragraph accordingly to allow for the addition of other results, specifically the age-stratified analysis that were added.

<u>*Page 11*</u>
Line 3 - is this in in-patient or in out-patient study populations? Or overall?

Response: All analyses beyond the first paragraph of the results and discussion respectively refer to exclusively in-patients. We have now clarified this by specifically mentioning outpatients in our discussion, to avoid ambiguity.

<u>*Page 11*</u>
Line 7 - this is important and perhaps should be mentioned earlier in the discussion. There is no mention of tourism seasonality in the discussion. Please include this.

Response: Thank you for this helpful suggestion. We have added a brief discussion of tourism seasonality for completeness. We note that its influence on RSV seasonality is likely limited in our setting, given that RSV predominantly affects very young children, who are less likely to travel, particularly when unwell. This rationale has been included succinctly in the Discussion.

<u>**Reviewer: 2**</u>
Dr. Mark Weatherall, University of Otago Wellington
Comments to the Author:
The manuscript reports a retrospective cohort study of children admitted to hospital in a single health jurisdiction with respiratory tract infection.
A weakness of the manuscript is the absence of a specific listed set of research aims. Based on the reporting of the results these research aims were likely: Identifying different characteristics of children admitted to hospital with a respiratory tract infection before and during or after COVID-19 became prevalent (described in Table 1), describing associations between different respiratory infection syndromes and disease and participant characteristics (left-hand columns of Table 2), describing associations between respiratory infection severity indicators and disease and participant characteristics (right-hand columns of Table 2), describe disease associations with seasonality (page 9 lines 25-40 and associated plots).

We thank Dr. Weatherall for the critical and relevant comments which helped us improve the manuscript in a meaningful way.

Response: We have added a list of specific research aims after the introduction and before the methods, to ensure that there is no confusion regarding the different research aims of the paper. The aims include:

1. To quantify pediatric RTI disease burden at St. Maarten Medical Center, describing care utilization for inpatients and outpatients
2. To assess, among inpatients with BioFire testing, the associations between individual respiratory pathogens and indicators of disease severity, using multivariable and age-stratified analyses.
3. To characterise the seasonality of pathogens associated with severity among inpatients, and to consider implications for the timing of preventive strategies where available.
4. To describe associations between respiratory infection syndromes and participant or clinical characteristics, and to compare these patterns across the pre- and post-COVID-19 periods.

In the statistical methods although logistic and linear regression are specified as methods it was difficult to see where linear regression was used as most results are reported as odds ratios.

Response: Linear regression was used exclusively for the association between pathogens and length of hospital stay pre- and post-COVID-19. We have now mentioned this explicitly in the Statistical analysis section of the Methods of the revised manuscript.

For Table 1 it might be better to separate it into two sections, one reporting scale variables and one reporting categorical variables, so that the two sections could have common column headers and it would be better to have a more fulsome description of the summary data for the scale variables e.g. include mean and SD. For this table there should be reporting of appropriate measures of association with a confidence interval and not just a P; and additionally, it is poor statistical practice to report boundary P values e.g. P<0.01. Reporting of P-values should be to appropriate precision as per the journal requirements.

Response: We have adjusted table 1 accordingly so that it will be easier to read. We included mean and SD for normally distributed variables and median and IQR (formerly median and range) for non-normally distributed variables. Also, we now report the confidence intervals for the continuous variables and the exact P-value instead of boundary P-values to ensure appropriate precision.

For some of the categorical variable associations it was unclear how this was handled e.g. if there were a set of mutually exclusive categories, then there should be an overall P-value for any evidence of a difference in proportions e.g. the 'Diagnosis' was likely mutually exclusive so there should be one P-value for association and odds ratios could be reported with reference to a baseline or reference level.

Response: Following the reviewer's suggestion, we have added odds ratios in Table 1 for the categorical variables and ensured that mutually exclusive categories were reassessed and handled appropriately so that only one overall P-value is presented in table 1.

It was also unclear how the scale variables were compared e.g. t-tests versus rank-based tests.

Response: T-tests were used for normally distributed continuous variables whereas Wilcoxon was used for non-normally distributed continuous variables. We have now added this explicitly in the description of Table 1.

For Table 2 it was unclear who the variables were chosen for the explanatory side of the logistic regression as these different slightly between the different response variables. This table would be better if there were numeric descriptors e.g. numerators/denominators and appropriate scale variable data descriptors per predictor in relation to response variable. Again, boundary P values are inappropriate.

Response: Table 2 shows the results of the logistic regressions for 5 different outcomes (URTI, LRTI, Viral wheeze, Oxygen requirement, and tachypnea/dyspnea). We used the same characteristic variables for each of the regressions (Age/gender), however, some pathogens were excluded for certain regression models when there were no patients with this pathogen who also presented with that specific outcome. We have now added this explicitly to the statistical analysis section of the methods for transparency. We also added exact P-values instead of boundary P-values, in accordance with the reviewer's suggestion.

In the weaknesses should be included Type I error inflation through repeated statistical testing and Type II error for detecting important associations limited by sample size.

Response: We have now added this weakness to the strengths and limitations paragraph of the discussion.

VERSION 2 - REVIEW

**Reviewer**	**1**
**Name**	**Izu, Alane**

Affiliation University of the Witwatersrand Johannesburg

Date 14-Nov-2025

COI

The title should specify what RTI disease

PG1

Line 8 - "long term risks" - dont think risk is the right word

Line 14 - what global burden?

PG 4

Line 48 "performed regression analyses to assess the association between presence of pathogen and ..."

Pg 5

Line 2 - as above "to assess the association between presence of pathogen and ..." - variable y is regressed on x.

Line 8 - Suggest rather saying "Wilcoxon rank-sum test and chi-sq test were used to assess the association between XX and XX. Linear and logistic regression was used to assess the association between various symptoms and timing (pre vs post covid) for symptoms with a normal distirbution and binomial distribution, respectively." its not entirely clear what these methods were used (I think objective 4? If so, can specify "to assess if respiratory syndromes and clinical characteristics where similar between patients that were hospitalised before and during COVID, ...")

Line 24 - "a p-value<0.05"

Line 40 and 41 - whats the definition of young and old children?

Line 49 - what does "compared in pairs" mean? What hypothesis was being tested?

Page 7

Line 1- Comparing pre-and post COVID-19 admission?

Line 22 - I would highlight the adjusted p-values as it may be misleading to highlight significant associations without adjusting for potential confounders.

Page 8

Line 22-25 - Agree with doing a stratified analysis of pre and post-COVID to assess association between oxygen and RSV. Including an interaction term would not assess the power. Suggest removing this.

Page 11

Line 19 - please use "associated" rather than "influenced" as this is an observational study.

Any adjustment for multiple testing?

**Reviewer**	**2**
**Name**	**Weatherall, Mark**
**Affiliation**	**University of Otago Wellington**
**Date**	**23-Oct-2025**
**COI**	

The authors have responded to the comments

VERSION 2 - AUTHOR RESPONSE

Reviewer: 2

Dr. Mark Weatherall, University of Otago Wellington
Comments to the Author:
The authors have responded to the comments

We thank Dr. Weatherall for his valuable comments in the previous review, and we are pleased to know that our answers to the comments are deemed satisfactory.

Reviewer: 1

Dr. Alane Izu, University of the Witwatersrand Johannesburg
Comments to the Author:

We thank Dr. Izu for the elaborate review and the additional comments to our work. We believe this detailed review will significantly help improve our work and fine-tune the manuscript.

The title should specify what RTI disease
We agree that the current title lacks specificity and can be improved. While we are hesitant to highlight a single pathogen, since we investigated a variety of common pathogens, we have added the word ‘respiratory’ to clarify the type of disease and the associated severity, thereby avoiding ambiguity.

Page 1
Line 8 - "long term risks" - dont think risk is the right word

We fully agree that ‘risks’ is not the most fitting word in this context, therefore we have changed it to “morbidity”.

Line 14 - what global burden?

Here we refer to the global disease burden experienced by children due to RTIs. We have clarified this by changing the wording to “the global disease burden”.

Page 4
Line 48 "performed regression analyses to assess the association between presence of pathogen and ..."

We agree that the suggested phrasing is more accurate and have changed the sentence accordingly.

Page 5
Line 2 - as above "to assess the association between presence of pathogen and ..." - variable y is regressed on x.

We have changed the sentence accordingly similarly to the sentence above.

Line 8 - Suggest rather saying "Wilcoxon rank-sum test and chi-sq test were used to assess the association between XX and XX. Linear and logistic regression was used to assess the association between various symptoms and timing (pre vs post covid) for symptoms with a normal distirbution and binomial distribution, respectively." its not entirely clear what these methods were used (I think objective 4? If so, can specify "to assess if respiratory syndromes and clinical characteristics where similar between patients that were hospitalised before and during COVID, ...")

These methods were indeed used for objective 4. We clarified the sentence based on the reviewer’s suggestion: “Wilcoxon rank-sum test and Chi-square test were used to assess the association between patient characteristics and pre- and post-COVID-19, for continuous and binary variables, respectively. Regression was used to assess the association between severity outcomes and period (pre- and post-COVID-19), where linear regression was used for length of hospital stay and logistic regression for all other outcomes.”

Line 24 - "a p-value<0.05"

We have changed this in the text accordingly.

Line 40 and 41 - whats the definition of young and old children?

“Young children” and “older children” are defined as ≤2 years and >2 years of age, respectively. This is defined in the methods page 5 line 18. We chose to not repeat this definition each time these subgroups are mentioned for conciseness and instead describe it clearly in the methods.

Line 49 - what does "compared in pairs" mean? What hypothesis was being tested?

With compared in pairs, we mean that we compared the proportion of coinfection of all pathogens to each other individually in pairs, as pathogens were not mutually exclusive. The hypothesis tested here was that the proportion of coinfection was higher in cases where adenovirus and/or parainfluenza was detected, compared to cases where rhino/enterovirus and/or influenza were tested. We have rephrased this sentence for clarify this point.

Page 7
Line 1- Comparing pre-and post COVID-19 admission?

We have changed this to “Comparing pre- and post-COVID-19 subsets of admissions…”

Line 22 - I would highlight the adjusted p-values as it may be misleading to highlight significant associations without adjusting for potential confounders.

All p-values presented in Table 2 are adjusted p-values, therefore the highlighted p-values are already adjusted for confounders.

Page 8
Line 22-25 - Agree with doing a stratified analysis of pre and post-COVID to assess association between oxygen and RSV. Including an interaction term would not assess the power. Suggest removing this.

We agree that this paragraph may be worded unclearly. What we meant was not that including the interaction term would directly assess the power, but rather that by including the interaction term we could confirm that this difference we saw pre- and post-COVID-19 was indeed due to time difference, and therefore not just because of a power difference. We have rephrased this so that our intention is more clear: “An additional logistic regression analysis, incorporating the interaction between time period and rhino/enterovirus as well as time period and RSV, was performed to determine if this difference was a true effect of time and not due to a power imbalance between time periods.”

Page 11
Line 19 - please use "associated" rather than "influenced" as this is an observational study.

We have changed this in line with the reviewer's suggestion.

Any adjustment for multiple testing?

No adjustments for multiple testing were applied as this study was exploratory in nature.

VERSION 3 - REVIEW

**Reviewer**	**1**
**Name**	**Izu, Alane**
**Affiliation**	**University of the Witwatersrand Johannesburg**
**Date**	**01-Dec-2025**
**COI**	

The authors have addressed all comments.
